# Volunteer Plants’ Occurrence and the Environmental Adaptability of Genetically Modified Fodder Corn upon Unintentional Release into the Environment

**DOI:** 10.3390/plants12142653

**Published:** 2023-07-15

**Authors:** Han-Yong Choi, Eun-Gyeong Kim, Jae-Ryoung Park, Yoon-Hee Jang, Rahmatullah Jan, Muhammad Farooq, Saleem Asif, Nari Kim, Ji-Hun Kim, Dohyeong Gwon, Seong-Beom Lee, Seung-Kyo Jeong, Kyung-Min Kim

**Affiliations:** 1 Department of Applied Biosciences, Kyungpook National University, Daegu 41566, Republic of Korea; seedsaler@naver.com (H.-Y.C.); mfarooqsr@gmail.com (M.F.); saleemasif10@gmail.com (S.A.); jennynari@hanmail.net (N.K.); kim960520@naver.com (J.-H.K.); pondelionn@naver.com (D.G.); dltks6954@naver.com (S.-B.L.); tmdry1221@gmail.com (S.-K.J.); 2 Coastal Agriculture Research Institute, Kyungpook National University, Daegu 41566, Republic of Korea; egk@knu.ac.kr (E.-G.K.); icd0192@korea.kr (J.-R.P.); uni@knu.ac.kr (Y.-H.J.); rehmatbot@yahoo.com (R.J.); 3 Crop Breeding Division, National Institute of Crop Science, Rural Development Administration, Wanju 55365, Republic of Korea

**Keywords:** GMO, corn, release, volunteer plants, weediness

## Abstract

The number of corn cultivars that have been improved using genetically modified technology continues to increase. However, concerns about the unintentional release of living-modified organisms (LMOs) into the environment still exist. Specifically, there are cases where LMO crops grown as fodder are released into the environment and form a volunteer plant community, which raises concerns about their safety. In this study, we analyzed the possibility of weediness and volunteer plants’ occurrence when GMO fodder corn grains distributed in Korea are unintentionally released into the environment. Volunteer plants’ occurrence was investigated by directly sowing grains in an untreated field. The results showed that the germination rate was extremely low, and even if a corn seed germinated, it could not grow into an adult plant and would die due to weed competition. In addition, the germination rate of edible and fodder grains was affected by temperature (it was high at 20 °C and 30 °C but low at 40 °C and extremely low at 10 °C), and it was higher in the former than in the latter. And the germination rate was higher in Daehakchal (edible corn grains) than in Gwangpyeongok (fodder corn grains). The environmental risk assessment data obtained in this study can be used for future evaluations of the weediness potential of crops and the development of volunteer plant suppression technology in response to unintentional GMO release.

## 1. Introduction

Crops are the most important cultivated plants globally for food production [1] and, among them, corn (*Zea mays* L.) is the most widely cultivated [2]. The genetic engineering of crops plays a crucial role in improving their nutritional value [3], increasing resistance to disease-associated stress, and enhancing tolerance to herbicides [4]. However, the use of genetically modified (GM) crops to produce food often faces controversy despite extensive safety measures being taken before their release and the lack of evidence [5] regarding concerns associated with them [6]. One of these concerns is the potential unintended impact on the environment due to the random integration of transgenes [7]. This phenomenon could cause changes in the DNA sequence, leading to the production of new proteins, the alteration of existing metabolites, the formation of unknown metabolites, and the unintentional destruction of genes [8].

With the continuous increase in the import volume of GM agricultural products, there is an increasing need to conduct risk assessments and evaluate the technology behind these products, as well as protecting the agricultural environment against unintentional environmental transfer [9]. In Korea, the self-sufficiency rate for grains (excluding rice, which is a food resource) is very low, and the dependence on overseas imports is high [10]. This rate is especially low for feed grains, which account for less than 5%, and corn and soybeans are mostly imported [11]. Corn, a major feed grain destined for livestock, is largely produced and exported by a few countries [12], and its production mainly depends on GM technology, which highlights the limitations Korea faces in meeting the demand for non-GM grain imports [13]. As the production, import, and distribution of GMOs continue to increase, controversy over their safety persists [14]. A prime example is a study of the chronic toxicity of the herbicide-resistant maize NK603 conducted in France in 2012 and whose experimental results are currently being discussed [15]. A study found that rats fed with a diet containing NK603 maize developed tumors and other health problems, leading to concerns about the safety of GMOs for food production [16]. Although GM foods are subjected to environmental risk assessments before being imported, public concerns regarding their safety and management still persist [17]. Among the potential negative impacts of GMOs on the environment are the disruption of the evolution of organisms, the destruction of biodiversity, weed infestation, the emergence of resistance in GM plants, the consequent emergence of mutations in pests or weeds, genetic transfer, and the alteration of soil microorganisms [18].

The unintentional release of GMOs into the environment has also been reported in Korea. According to the results of environmental monitoring conducted by the Ministry of the Environment, such incidents occurred in 33 cultivated areas (7 with rapeseed, 19 with corn, and 7 with cotton) in 2012 [19]. There is a possibility of the unintentional release of GMOs into the environment and the consequent spread of genetic contamination [20].

Using genetic engineering technology, the biotechnology industry is currently developing and producing GMOs that are expected to create high-added-value products. However, there are ongoing controversies over their safety, and international efforts are being made to ensure biosecurity [21,22]. On 11 September 2003, the Protocol on Biosafety officially came into effect [23], and it is now necessary to develop and establish a scientific safety evaluation technology and assessment system that is suitable for the situation in Korea.

Instances of unintentional GMO release have been reported during field monitoring activities [20], and on-site removal efforts have been carried out. However, given that the majority of GMOs are herbicide tolerant, it was difficult to remove them from the affected sites [24].

A rapid response is also necessary for the increase in the dormancy rate due to hybridization [25]. Therefore, it is crucial to conduct a risk analysis of the unintentional release of GMOs into the natural environment and develop technologies and strategies to prevent weediness and inhibit the occurrence of volunteer plants from releasing GMOs is an urgent task [20]. This research aimed to collect the basic information required for achieving this task and to determine the feasibility of technologies based on such information.

## 2. Results

### 2.1. Analysis of the Germination Rate of Edible and Fodder Corn Grains Based on Temperature

**Figure 1:**
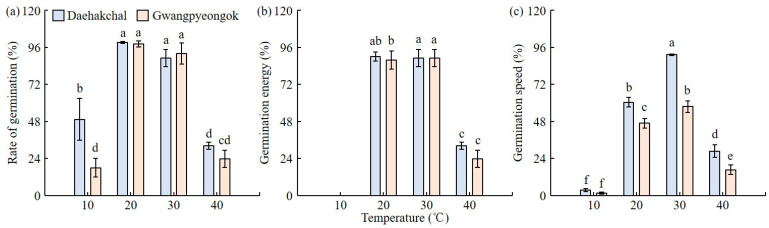
Germination rate analysis of edible and fodder corn grains by temperature. Rate of germination (**a**), germination energy (**b**), and germination speed (**c**) of edible corn (Daehakchal) and fodder corn (Gwangpyeongok) grains by temperature. (**a**–**c**) were all significantly lower at 10 °C and 40 °C. The means denoted by the same letter are not significantly different (*p* < 0.05) as evaluated by Duncan’s multiple range test (DMRT).

The germination rate (%) of Daehakchal was 49.3 ± 13.6% at 10 °C and 99.3 ± 1.2% at 20 °C (Figure 1). And the germination rate was 89.0 ± 5.6% at 30 °C and 32.3 ± 2.3% at 40 °C. The germination rate of Gwangpyeongok was 18.0 ± 6.1% at 10 °C and 98.0 ± 2.0% at 20 °C. And it was 92.0 ± 6.9% at 30 °C and 23.7 ± 5.5% at 40 °C. The germination energy (%) of the Daehakchal grains that did not germinate for 3 days at 10 °C. At 20 °C, germination energy was 90.0 ± 3.0% for 3 days. And it was 89.0 ± 5.6% at 30 °C and 32.3 ± 2.3% at 40 °C. The germination energy (%) was zero for both Daehakchal and Gwangpyeongok under 10 °C for three days, indicating that no seeds germinated, and the rate was 87.7 ± 5.9% at 20 °C. And it was 89.0 ± 5.6% at 30 °C and 23.7 ± 5.5% at 40 °C. The germination speeds of the Daehakchal grains at the four temperatures were 3.6 ± 0.9%, 60.5 ± 3.0%, 91.2 ± 0.6%, and 28.7 ± 4.1%. Those of the Gwangpyeongok grains were 1.5 ± 0.6%, 46.9 ± 3.1%, 57.6 ± 3.8%, and 16.7 ± 3.0%.

### 2.2. Comparison of Agricultural Traits between Edible and Fodder Corn

**Figure 2:**
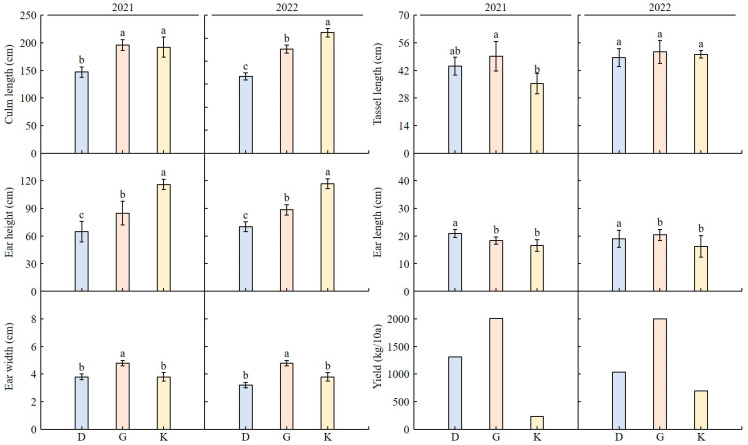
Analysis of major agronomic traits of edible (Daehakchal) and fodder (Gwangpyeongok) corn grains: culm length (cm), tassel length (cm), ear height (cm), ear length (cm), ear width (cm), and yield (kg/10a). The agricultural trait data indicated similar results for 2 years (2021 and 2022). The mean values denoted by the same letter are not significantly different (*p* < 0.05) as evaluated by Duncan’s multiple range test (DMRT). D: Daehakchal; G: Gwangpyeongok; K: KM5.

In 2021 and 2022, KM5 had the longest culm length, while Daehakchal had the shortest (Figure 2). In 2021, Gwangpyeongok had a longer tassel length than KM5; in 2022, there was no difference in length. In both 2021 and 2022, KM5 had the longest ear height, while Daehakchal had the shortest. As for ear length, Daehakchal was the longest in both 2021 and 2022. In both 2021 and 2022, Gwangpyeongok had the widest ear width and the highest yield.

### 2.3. Viability Analysis of Volunteer Plants Derived from the GM Fodder Corn

In 2021 and 2022, a total of 28 and 39 plants, respectively, sprouted and grew spontaneously from crushed corn grains, whose germination rates during these two years were 0.01% and 0.07%, respectively (Table 1). However, all the volunteer plants derived from the corn grains died due to competition with weeds. The germination rate of the crushed corn grains was considerably lower than that of the normal corn grains.

### 2.4. Analysis of the Corn Grain Dormancy Rate and Environmental Conditions at the Loading Ports

**Figure 3:**
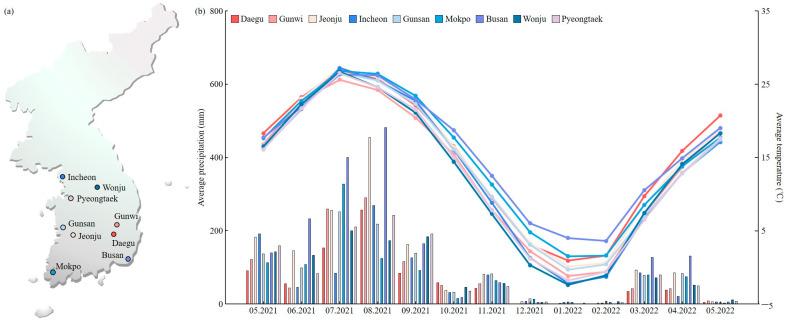
Comparison of environmental conditions in the three Korean regions examined (Daegu, Gunwi, and Jeonju) where the edible and fodder corn grains were buried and in six important port areas. (**a**) Location of each region on the map of Korea. Daegu, Gunwi, and Jeonju regions where the field test was conducted and Korea’s major import/export ports (i.e., Incheon, Gunsan, Mokpo, Busan, Wonju, and Pyeongtaek). (**b**) Monthly average temperature and precipitation in each region.

Most grains germinated at a rate of 99.7%, and their dormancy rate was 0%. Of the first 100 grains sampled, 97 germinated and 3 did not. To analyze the dormancy rate, the germination rate was investigated using three non-germinated seeds, but they all rotted without germinating. In Daegu, Gunwi, and Jeonju, the dormancy rate of the buried corn grains was 0%. In addition, the average monthly temperature and precipitation in the three regions examined and in the major import and export ports of Korea (i.e., Incheon, Gunsan, Mokpo, Busan, Wonju, and Pyeongtaek) were investigated over a period of 1 year (Figure 3). The results did not reveal significant differences in these parameters among the regions.

### 2.5. Comparison of Weediness in the Edible and Fodder Corn

**Figure 4:**
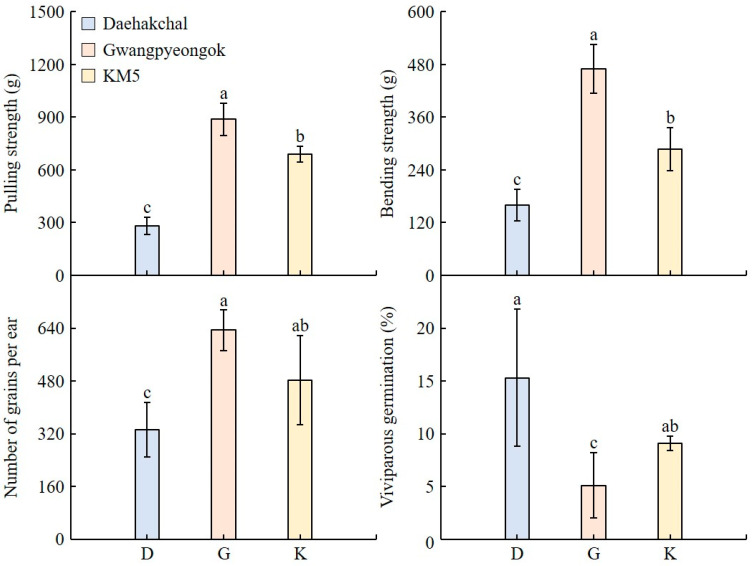
Analysis of weediness in edible (Daehakchal) and fodder (Gwangpyeongok) corn grains. Weediness was assessed based on the shattering and viviparous germination of grains using the corn ear. The fodder corn grains required more force to shatter them than the edible corn grains and had lower viviparous germination rates. Mean values denoted by the same letter are not significantly different (*p* < 0.05) as evaluated by Duncan’s multiple range test (DMRT). D: Daehakchal; G: Gwangpyeongok; K: KM5.

The pulling strengths (g) were highest for Gwangpyongok, requiring the most force to detach the grains from the ear, while Daehakchal had the lowest pulling strength, making it relatively easy to detach the grains from the ear (Figure 4). Among the three samples, Gwangpyeongok had the highest bending strength (g), followed by KM5 and Daehakchal. Both pulling and bending strengths were higher in Gwangpyeongok than in Daehakchal; therefore, the degree of shattering of the former cultivar was lower. The number of grains per ear was the same for Gwangpyeongok and KM5, while Daehakchal had the lowest number. The viviparous germination rate (%) was the same for Daehakchal and KM5, with Gwangpyeongok having the lowest viviparous germination rate.

## 3. Discussion

Maize is one of the most important crops and the main staple crop used for human consumption and as livestock feed [26]. GM technology has been the driving force behind the increase in the yield of maize and the improvement of its agricultural traits [27]. The development of GM technology has led to the breeding of transgenic maize with improved traits [28]. Safety assessment procedures for GM crops are primarily based on the analysis of their composition, which specifically targets nutritionally relevant compounds [29]. However, this targeted analysis is limited in terms of detecting the unintended effects of GM crops [30]. Thus, concerns about the unintentional release of GM grains into the environment and their impact on it are widespread among researchers and the public [31]. Comprehensive environmental risk assessments are conducted worldwide to address these concerns [32]. However, laboratory research alone is limited in mitigating the reservations about GM crops expressed by environmental organizations and the public [33]. In this study, laboratory and field experiments were performed to analyze the environmental adaptability and weediness of GM corn used for livestock feed upon unintentional release into the environment. In Korea, GM corn is used as fodder for livestock and not for human consumption.

We cultivated one foreign (KM5) and two Korean (Daehakchal and Gwangpyeongok) corn cultivars in the same location/environment for 2 years (2021 and 2022) and investigated their environmental adaptability, weediness, and major agricultural traits. The culm length was the longest in Gwangpyeongok and KM5 in 2021 and 2022, respectively. In 2021, the tassel length (cm) did not differ between Daehakchal and Gwangpyeongok and was shorter in KM5 than in Gwangpyeongok. In 2022, the three cultivars showed no differences in tassel length. As for ear height, the longest and shortest values were detected in KM5 and Daehakchal, respectively, in both years. The ear length was the longest in Daehakchal in both 2021 and 2022, and no differences were observed between Gwangpyeongok and KM5. The ear width was the longest in Gwangpyeongok in both years, and Daehakchal and KM5 showed similar values. As for the yield, the highest and lowest values were detected in Gwangpyeongok and KM5, respectively, in both 2021 and 2022. Overall, differences in these agricultural traits were observed among all corn cultivars, but there was no clear difference between the domestic and foreign cultivars. The highest germination rates were achieved at 20 °C and 30 °C and the lowest were achieved at 10 °C for both of the Korea cultivars, and the values were similar between them. The germination energy was also the highest at 20 °C and 30 °C and the lowest at 10 °C, and the values were also similar between the two cultivars. The highest germination speed was detected at 30 °C for both cultivars, followed by 20 °C, 40 °C, and 10 °C in this order. This parameter was higher in Daehakchal than in Gwangpyeongok. Therefore, even in the event of unintentional release into the environment, the germination rate of the grains would be extremely low at high temperatures above 40 °C or low temperatures below 10 °C [34]. Even if GM corn grains are unintentionally released into the environment, the concern regarding environmental contamination from GM corn grains would be low in countries with four distinct seasons or with prolonged periods of extreme temperatures, such as temperatures above 40 °C or below 10 °C.

The volunteer plants’ potential in the natural environment due to unintentional release during the distribution of GM corn feed was investigated by directly sowing crushed corn grains in Kyungpook National University GM fields. The proportion of grains that grew into plants was 0.01% in 2021 and 0.07% in 2022. This was extremely low compared to the proportion of plants grown from normal corn grains. It is therefore suggested that if GM seeds are imported or exported in the form of crushed grain rather than whole grain, it is possible to suppress volunteer plants’ growth. The environmental conditions at major import ports were investigated to analyze the potential occurrence of weediness during the unintentional release of GM corn grains into the environment. Approximately 100 g of corn grains were buried in three regions of Korea (Daegu, Gunwi, and Jeonju), and their dormancy rate was investigated by conducting monthly samplings for 2 years. The results showed that the dormancy rate of the grains was 0%. In addition, it was revealed that the average temperature and precipitation at Daegu, Gunwi, Jeonju, and the import ports (i.e., Incheon, Gunsan, Mokpo, Busan, Wonju, and Pyeongtaek) were not significantly different from each other. Therefore, even if the unintentional release of GM grains occurred during import operations, the growth rates would be similar to those observed in Daegu, Gunwi, and Jeonju. In addition, if the unintentional release occurred due to spillage or burial during transportation to major GM grain distribution areas, the discovery of germinated grains would be highly unlikely. When released into the environment, which may occur due to unintentional actions during ear harvesting from GM plants, to analyze weediness, we investigated the shattering and viviparous germination of the grains. The shattering of grains is a major characteristic of grain dispersal, and most wild plants or weeds have a high shattering of grains [35]. In this study, the pulling and bending strengths of Gwangpyeongok were higher than those of Daehakchal, and the shattering of grains was greater in the latter cultivar. In addition, the viviparous germination rate was higher in Daehakchal than in Gwangpyeongok. Therefore, the possibility of weediness of the grains due to unintentional release into the environment was low, in line with Joen and Park [36].

The present study attempted to evaluate the environmental risk of the unintentional release of corn grains into the environment. The results obtained can be used to establish guidelines for the environmental risk assessment of GM agricultural products. By analyzing the adaptability of the corn grains to the external environment, it was determined that even if GM corn grains were unintentionally released into the natural environment, there would be no cause for concern about environmental pollution associated with GM plants because of the grains’ low dormancy rate and low weed competition in natural fields.

## 4. Materials and Methods

### 4.1. Corn Cultivation and Analysis of Agricultural Traits

To investigate the main agricultural traits of fodder and edible corn, seeds were sown in the Gunwi GM field at Kyungpook National University at a planting distance of 90 cm × 30 cm in 2021 and 2022. The seeds used in the experiment belonged to the Korean cultivars Daehakchal and Gwangpyeongok, the former edible and the latter used as fodder in livestock feed blends. KM5 is a corn cultivar bred for export purposes. KM5 is highly resistant to diseases and adaptable to both tropical and subtropical regions. Mulching was performed for weed control after creating 120 cm-wide ridges, and the seeds of each cultivar were sown at intervals of 30 cm per ridge. The investigated agricultural traits at 10 times were culm length, tassel length, ear height, ear length, ear width, and yield. Culm length was measured as the distance from the ground to the top leaf collar, and tassel length was measured as the distance from the top leaf collar to the central spike. Ear height and length were defined as the distance from the ground to the highest node with an ear and the length of the ear excluding the silk, respectively. Ear width was measured as the diameter of the central part of the ear.

### 4.2. Germination Rate Analysis of Seeds for Fodder and Consumption by Temperature

To investigate the vitality of the corn seeds depending on temperature, germination was induced in the Daehakchal and Gwangpyeongok cultivars at 10 °C, 20 °C, 30 °C, and 40 °C. For each temperature and cultivar, 50 seeds were placed on a 90 mm × 15 mm Petri dish (SPL Life Science, Pocheon, Republic of Korea), which was covered with filter paper (Cat No 1002 090, Whatman Int’l Ltd., Maidstone, UK) and soaked in distilled water. The experiments were repeated three times to induce germination. The germination rate, germination energy, and germination speed at different temperatures were then determined. The germination rate (%) was defined as the ratio of germinated seeds to the total number of seeds (germination rate = [total number of grains germinated/total number of grains] × 100), and germination energy (%) was defined as the ratio of grains that germinated within 3 days to the total number of grains (germination energy = [total number of grains up to 3 days after test/total number of grains] × 100). The germination speed was calculated based on the number of days that had elapsed after planting the grains using the following equation: germination speed = N_1_/D_1_ + (N_2_ − N_1_)/D_2_ + (N_3_ − N_2_)/D_3_ + … + (N_j_ − N_i_)/D_j_, where N is the number of germination seeds on the counting date and D is the number of days.

### 4.3. Analysis of the Occurrence of Volunteer Plants Resulting from the Unintentional Release of GM Corn Feed

To analyze the environmental release of GM feed due to unintentional release during the distribution of GM corn feed, we directly sowed the crushed grains, a commercially available product, in the Gunwi field. The crushed GM corn grains are sold, however, during the manufacturing process, and the seeds may not be evenly crushed, so there is a possibility that the seeds’ embryos are preserved, leading to germination. Therefore, we analyzed the rate of volunteer plants’ occurrence caused by unintentional release using GM corn feed. A total of 75 kg of crushed corn grains was sown in a field with an area of 750 cm^2^. To reproduce the natural environmental conditions as much as possible, the field was not subjected to any artificial treatments, such as irrigation or fertilization. In addition, the level of weed competition in the field was assessed to evaluate the potential for volunteer plants’ growth.

### 4.4. Analysis of Weediness Potential in the Edible and Fodder GM Corn Grain

The shattering and viviparous germination of Daehakchal (edible corn cultivar), Gwangpyeongok (fodder corn cultivar), and KM5 (fodder corn cultivar) corn grains were investigated to analyze the potential weediness due to the unintended release of GM grains into the environment during distribution. To determine the shattering levels, the pulling and bending strengths were measured using a strain gauge (IMADA, DPS-5, Japan). The pulling strength was measured parallel to the axis of the corn ear, and the bending strength was measured at the moment when the seed shattered as the force was applied in the direction perpendicular to the ear’s axis. Shattering was measured by conducting 10 repetitions on each corn ear. To assess viviparous germination, the husks were removed from the harvested corn, wrapped in a damp cloth, and kept at a humidity of 80% and a temperature of 30 °C for 15 days, during which the germination rate was measured. This assessment was conducted three times for each cultivar.

### 4.5. Investigation of the Dormancy Rate of Corn Grains by Region

To analyze the potential weediness of volunteer plants that may occur due to unintentional release into the environment, such as accidental seed release during the distribution of GM corn feed, 100 corn grains for each cultivar (Daehakchal and Gwangpyeongok) were buried at the depth of 5 cm in the soil environments of three regions of Korea; Daegu, 5°53′40.88″ N 128°36′47.34″ E; Gunwi, 36°6′41.54″ N 128°38′26.17″ E; and Jeonju, 35°49′53.33″ N 127°03′50.15″ E). The experiment was carried out in triplicate. The seeds were excavated at 1-month intervals for 24 months, and their germination rate was determined to analyze the dormancy rate. Soil moisture and temperature were also measured in triplicate using a Takeme-10EC meter (Takeme-10EC, Veinasa, Mianyang, China).

### 4.6. Investigation of Average Temperature and Precipitation in Areas located near the GM Seed Distribution Pathways

To analyze the potential for germination in the event that GM seeds are unintentionally released during distribution, the monthly average temperature and precipitation in regions crossing the major distribution pathways in Korea were investigated over a period of 1 year. GM crop cultivars in Korea depend on imports from abroad. They are imported from ports and transported to regions where they are needed. Unintentional release may occur during transport. Therefore, the average climate of the port surroundings and three regions (Daegu, Gunwi, and Jeonju) were investigated. Specifically, differences in these parameters were investigated in the port areas of Incheon, Gunsan, Mokpo, Busan, Wonju, and Pyeongtaek as well as in the Daegu, Gunwi, and Jeonju regions where the GM grains were buried.

### 4.7. Statistical Analysis

The average and standard deviation values of all data measured in this study were calculated at least three times and statistically analyzed using SPSS statistical software (IMMSPSS Statistics, version 22; IBMSPSS Statistics, version 26, Redmond, DC, USA). All data were presented as the mean ± standard deviation and analyzed through a *t*-test. One-way ANOVA and Duncan’s multiple range test were used to determine statistically significant differences (*p* < 0.05) in the average values among three or more groups.

## 5. Conclusions

In this research, the germination rate, major agricultural traits, volunteer plants’ occurrence, dormancy, shattering, and viviparous germination of grains were investigated to assess the environmental impact of GM corn grains unintentionally released into the environment. The germination rate was meager at a low temperature of 10 °C and a high temperature of 40 °C. The occurrence of volunteer plants due to the sowing of GM fodder corn in the natural field was extremely low, and even if they emerged, none of them would survive because of weed competition. The dormancy rate of the buried corn grains was also extremely low. In addition, the analysis of the weediness of edible and fodder corn grains revealed that the shattering and viviparous germination were lower in the fodder grains than in the edible grains. Therefore, even if GM corn grains are unintentionally released into the natural environment, it is suggested that the degree of concern about ecological contamination of the natural environment is lower. The results of this research can be used to establish guidelines for the environmental risk assessment of GM crops, specifically of GM corn, and to develop technology that suppresses volunteer plants’ occurrence to address the potential unintentional release of GMOs into the environment in the future.

## Figures and Tables

**Table 1:** Analysis of volunteer plants derived from GM fodder corn in natural conditions.

Year	Amount of Sowing (kg)	100-Grain Weight Conversion Quantity	Number of Plants	Rate of Germination (%)
2021	75	214,300	28	0.01
2022	20	57,100	39	0.07

## Data Availability

Not applicable.
